# Pan-cancer analysis reveals potential immunological and prognostic roles of METTL7A in human cancers

**DOI:** 10.1038/s41598-024-54255-x

**Published:** 2024-02-12

**Authors:** Bin Wang, Jingjing Jiang, Danju Luo, Xiong Wang

**Affiliations:** 1 Department of Laboratory Medicine, Tongji Hospital, Tongji Medical College, Huazhong University of Science and Technology, Wuhan, China; 2 Department of Obstetrics and Gynecology, Tongji Hospital, Tongji Medical College, Huazhong University of Science and Technology, Wuhan, China; 3 Department of Pathology, Union Hospital, Tongji Medical College, Huazhong University of Science and Technology, Wuhan, China

**Keywords:** Pan-cancer, Bioinformatic analysis, METTL7A, Expression, Prognosis, Cancer, Genetics

## Abstract

Methyltransferase-like protein 7A (METTL7A) is an m6A RNA methyltransferase that has been linked to cancer prognosis and drug resistance. However, a comprehensive analysis of METTL7A is lacking. The expression of METTL7A, prognostic performance, correlation with microsatellite instability (MSI), tumor mutational burden (TMB), and immune infiltration was investigated in The Cancer Genome Atlas (TCGA). Immunohistochemistry staining was applied to detect METTL7A in 6 tumors. METTL7A was significantly decreased in 19 cancers in TCGA including LUAD. Alterations of METTL7A include amplification and mutation, and epigenetic alterations revealed increased promoter methylation may result in down-regulation of METTL7A in LUAD. We also found that METTL7A was linked to both TMB and MSI in LUAD. METTL7A was increasingly correlated with invasive immune cells, while being negatively associated with Macrophages M0, Mast cells activated, activated memory CD4 T cells, CD8 T cells, and follicular helper T cells in several tumors. Additionally, METTL7A showed similar correlation with immune therapy-related genes across cancers. Our biological validation found that the protein levels of METTL7A were down-regulated in breast cancer (BRCA), endometrioid cancer (UCEC), colon cancer (COAD), prostate cancer (PRAD), and kidney clear cell carcinoma (KIRC), as detected by immunohistochemistry staining. Overall, our work indicates that METTL7A may serve as promising diagnostic and prognostic indicator of LUAD, and our work sheds light on the potential immunological and prognostic roles of METTL7A in human cancers.

## Introduction

Cancer remains the leading cause of death and a significant public health concern, posing a threat to both our health and financial liability, as well as our quality of life^1^. In China alone, it is estimated that in 2022, there would be 4,820,000 new cancer cases, and 3,210,000 cancer-related deaths, with lung cancer being the most frequent type of cancer^2^. Today, cancer treatment strategies typically involve surgery, targeted therapy, chemotherapy, radiotherapy, and immunotherapy. Targeted therapy and immunotherapy, such as immune checkpoint blocking therapy, have become prominent cancer treatments. However, patient response to these therapies varies significantly, and the survival rate of patients is still unsatisfactory^3^. Therefore, there is an urgent need to identify novel diagnostic biomarkers and new therapeutic strategies^4^. Despite the vast heterogeneity of cancers across different tissues, there are numerous similarities in cancer initiation and progression. Therefore, it is valuable to explore the expression pattern, clinical and therapeutic significances of key genes during the initiation and development of various cancers through pan-cancer research^5^. With the advancement of the public databases, pan-cancer bioinformatics analysis has become available and widely used to discover potential therapeutic targets.

N6-methyladenosine (m6A) is a critical mRNA modification for physiological and carcinogenic processes, involving m6A writers, readers, and erasers^6^. Several methyltransferases, including METTL3/14/16, RBM15/15B, CBLL1, ZC3H3, KIAA1429, VIRMA, and WTAP, participate in this process, and m6A-related genes have been reported to be correlated with the clinical diagnosis of cancer^7^. Methyltransferase-like protein 7A (METTL7A), a member of the methyltransferase-like protein family, was initially considered as lipid metabolism-related integral membrane protein located in the endoplasmic reticulum that promotes lipid droplet formation^8^. However, in the last decade, METTL7A has been found to be involved in the carcinogenesis of various tumors, acting as both oncogene and tumor suppressor, depending on the type of cancer^9^. For example, METTL7A acted as a protective factor for lung adenocarcinoma (LUAD)^10^, whereas it played a tumor suppressive role in primary thyroid cancers (THCA) and hepatocellular carcinoma^11,12^. Conversely, METTL7A was upregulated in methotrexate-resistant choriocarcinoma cells, the increased METTL7A promoted cell viability, clonogenesis, suppressed apoptosis, and reduced the production of reactive oxygen species, indicating an oncogenic role in choriocarcinoma^13^. While METTL7A generally functions as a tumor suppressor, its underlying mechanisms and clinical implications across cancers remain unclear. An extensive study of the transcriptome and epigenome related to METTL7A could shed light on its varying expression in different types of cancer, and suggest possible mechanisms of its action.

We performed a pan-cancer integrated analysis to investigate the role of METTL7A. The transcriptome and methylation datasets from The Cancer Genome Atlas (TCGA) were used to explore expression profile of METTL7A across human cancers. We evaluated the prognostic value of METTL7A, genetic alteration, correlation with clinical features, microsatellite instability (MSI), tumor mutational burden (TMB), immune cell infiltration, and immune therapy-associated genes across 33 types of cancers in TCGA. We also performed biological validation using immunohistochemistry staining to detect METTL7A protein levels in several tumors.

## Materials and methods

### Data processing

TCGAbiolinks R package (v2.25.3) was used to download TPM matrix, clinical information, TMB, and methylation matrice for the 33 types of cancers in TCGA (https://portal.gdc.cancer.gov/) with the GDCquery, GDCdownload, and GDCprepare functions^14^. The expression matrix was generated using log_2_(TPM + 1) and prepared using TCGAplot R package (v4.0.0)^15^, we did not perform data quality checks. The methylation probe was annotated using ChAMP R package (v2.24.0), and the promoter probes were defined with “feat.cgi =  = ’TSS1500-island’” parameter^16^. The CIBERSORT immune fractions were downloaded from TCGA database. The Microsatellite Instability (MSI) data was downloaded with cBioPortalData R package (v2.6.1) by accessing studies from the cBio Cancer Genomics Portal^17^.

The ggpubr R package (v0.4.0) was utilized to compared and plot METTL7A expression between different tissues. TCGA tumors with sufficient normal samples for Area Under the Curve (AUC) analysis were further used for AUC analysis grouped by the expression of METTL7A. The pROC R package (v1.18.0) was applied to evaluate the diagnostic potential of METTL7A in distinguishing tumors from normal samples, and the ROC curve was plotted and the AUC with 95% CI was presented^18^.

### Immunohistochemistry

The surgical specimens from Breast Invasive Carcinoma (BRCA), Uterine Corpus Endometrial Carcinoma (UCEC), Colon adenocarcinoma (COAD), Prostate adenocarcinoma (PRAD), Kidney renal clear cell carcinoma (KIRC), and LUAD were fixed, routinely dehydrated, paraffin-embedded, and sectioned at 4 μm. The primary METTL7A antibody (Bioswamp, Wuhan, Chinae, Catalog: PAB35752) was used for immunohistochemical staining by the Envision two-step method.

### Prognosis potential analysis of METTL7A

The OS was assessed to evaluate the prognosis potential analysis of METTL7A. The survminer (v0.4.9) and survival (v3.3-1) R packages were utilized to perform log-rank test and chart the KM plot for data grouped by expression of METTL7A.

We used survival and forestplot (v3.1.1) R packages to perform Cox analysis to ascertain the connection between METTL7A expression and survival across 33 types of cancers in TCGA.

### Correlation of METTL7A expression with mutational tumor heterogeneity

The correlation between METTL7A and TMB or MSI was assessed using Pearson correlation analysis, and the radar plot was drawn with the fmsb R package (v0.7.5).

### Relationship between METTL7A expression and immunity

The list of genes was downloaded from the TISIDB (http://cis.hku.hk/TISIDB/index.php)^19^. The correlation between METTL7A and immune related genes, immune infiltration was examined using Pearson correlation analysis. The correlation was plotted with ggplot2 R package (v3.3.5).

The estimate R package (v1.0.13) was utilized to compute the immune, stromal, and estimate scores for each specimen. The correlation between METTL7A and these scores was assessed using Pearson correlation analysis, and was plotted using ggplot2 (v3.3.5) and ComplexHeatmap (v2.10.0) R packages.

### Biological significance of METTL7A in tumors

Cancer specimens were grouped into METTL7A high and low subgroups. Differentially regulated genes were determined with limma R package (v3.46.0). All genes were ordered by decreasing according to the fold change (high-risk vs low-risk group), and these genes were used as input for GESA-GO and GSEA-KEGG analysis performed with org.Hs.eg.db (v3.14.0), clusterProfiler (v4.2.2) R packages and KEGG database (https://www.kegg.jp/kegg/)^20^. The top five terms were plotted with enrichplot R package (v1.14.2).

### Ethical approval

Ethics approval and consent to participate this study involving human participants were reviewed and approved by the Ethics Committee of Tongji Hospital, Tongji Medical College, Huazhong University of Science and Technology. The patients provided their written informed consent to participate in this study. All experiments were performed in accordance with the declaration of Helsinki.

## Results

### Pan-cancer expression, diagnostic, and prognostic analyses of METTL7A

**Figure 1:**
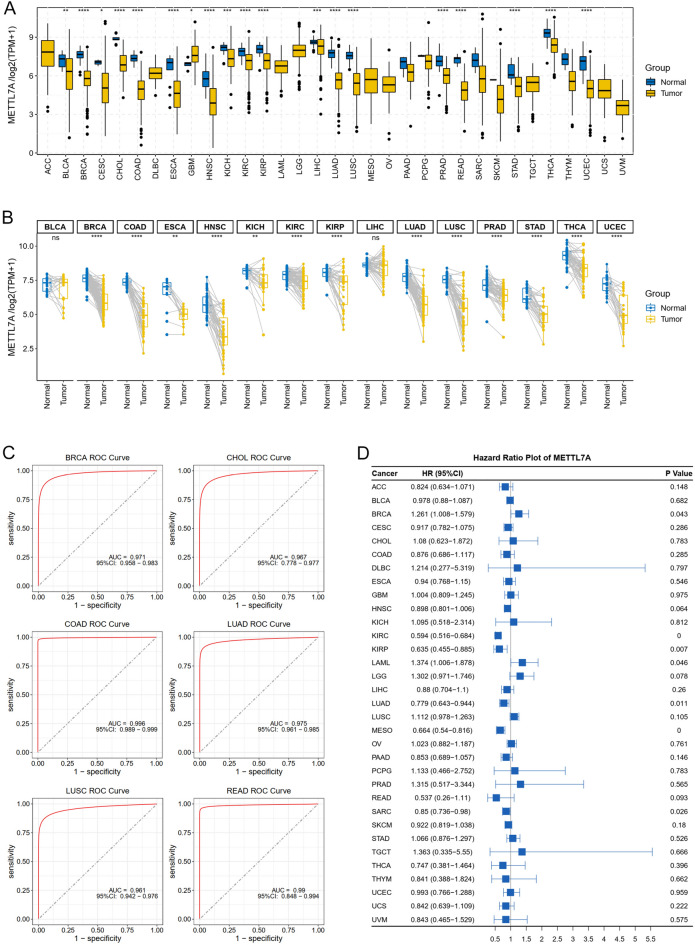
Pan-cancer expression analysis of METTL7A. (**A**) Boxplot showed the expression of METTL7A between tumor and normal specimens across 33 types of tumors in TCGA. Data was shown as log_2_(TPM + 1). (**B**) Paired boxplot showed the expression of METTL7A between matched tumor and paired normal specimens across 15 types of tumors in TCGA. Data was shown as log_2_(TPM + 1). (**C**) METTL7A showed AUC values > 0.95 in several tumors, including BRCA, CHOL, COAD, LUAD, LUSC, READ. (**D**) Pan-cancer Cox proportional risk model analysis of METTL7A across 33 types of tumors in TCGA. Wilcoxon test was used to compare the expression of METTL7A between normal and tumor groups. *P < 0.05, **P < 0.01, ***P < 0.001, ****P < 0.0001. ns meant no significant difference.

The expression of METTL7A across 33 types of tumors in TCGA was evaluated by comparing each tumor with its controls (non-tumor samples). Considerable differences in METTL7A expression were observed across 19 types of tumors. METTL7A was remarkably decreased in bladder urothelial carcinoma (BLCA), cervical squamous cell carcinoma and endocervical adenocarcinoma (CESC), BRCA, COAD, cholangio carcinoma (CHOL), esophageal carcinoma (ESCA), kidney chromophobe (KICH), head and neck squamous cell carcinoma (HNSC), KIRC, kidney renal papillary cell carcinoma (KIRP), LUAD, liver hepatocellular carcinoma (LIHC), lung squamous cell carcinoma (LUSC), rectum adenocarcinoma (READ), PRAD, stomach adenocarcinoma (STAD), THCA, and UCEC, while was only increased in glioblastoma multiforme (GBM) (Fig. 1A). We also explored METTL7A expression in paired tissues from 15 types of tumors whose matched sample size more than 20. Decreased expression of METTL7A was found in 13 out of 15 types of tumors with matched specimens compared with its paired controls (Fig. 1B). These data indicate that METTL7A was decreased across numerous TCGA tumors. Combined with analysis between tumor and paired (unpaired) normal tissues, we found that METTL7A was significantly down-regulated in 13 types of tumor from TCGA, including BRCA, COAD, ESCA, HNSC, KICH, KIRC, KIRP, LUAD, LUSC, PRAD, STAD, THCA, and UCEC.

We explored the diagnostic effectiveness of METTL7A and revealed that it exhibited AUC values greater than 0.95 in six different tumor types, including BRCA, CHOL, COAD, LUAD, LUSC, and READ (Fig. 1C). Cox regression analysis revealed that METTL7A acted as a beneficial factor in KIRC, KIRP, LUAD, Mesothelioma (MESO) (Fig. 1D). These results suggested that METTL7A had diagnostic and prognostic potentials in LUAD.

**Figure 2:**
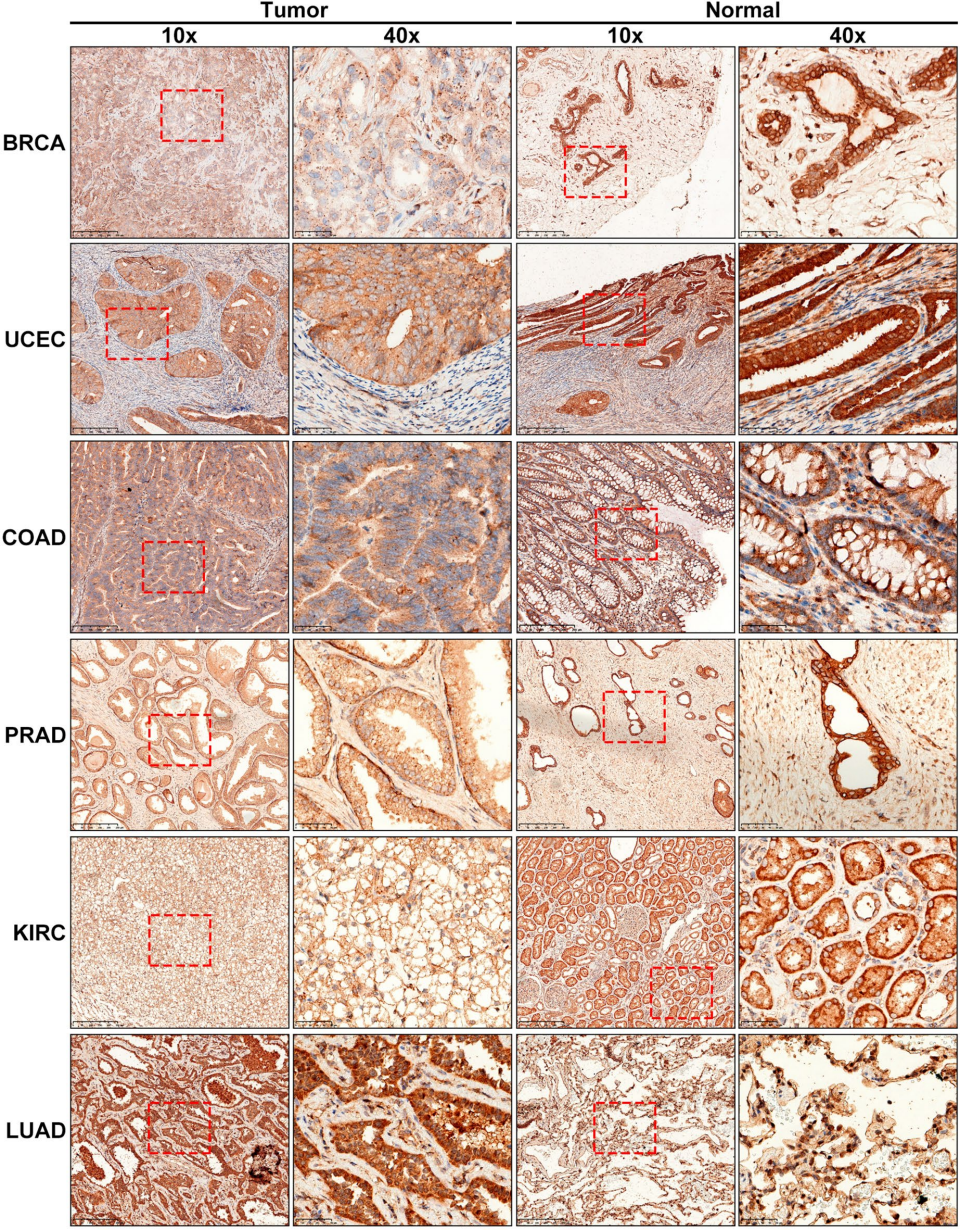
Immunohistochemical staining of METTL7A in human cancers. The left panel showed 10 × folds and enlarged 40 × folds immunohistochemical staining of METTL7A in tumor samples. The right panel showed 10 × folds and enlarged 40 × folds immunohistochemical staining of METTL7A in normal samples.

To examine the protein levels of METTL7A in tumors, we performed immunohistochemical staining of METTL7A in 6 types of tumor, including BRCA, UCEC, COAD, PRAD, KIRC, and LUAD (Fig. 2). The results showed that the protein levels of METTL7A was decreased in BRCA, UCEC, COAD, PRAD, and KIRC compared with the adjacent normal tissues, while no significant difference was observed in LUAD.

### Pan-cancer correlation analysis of METTL7A and clinical phenotypes

**Figure 3:**
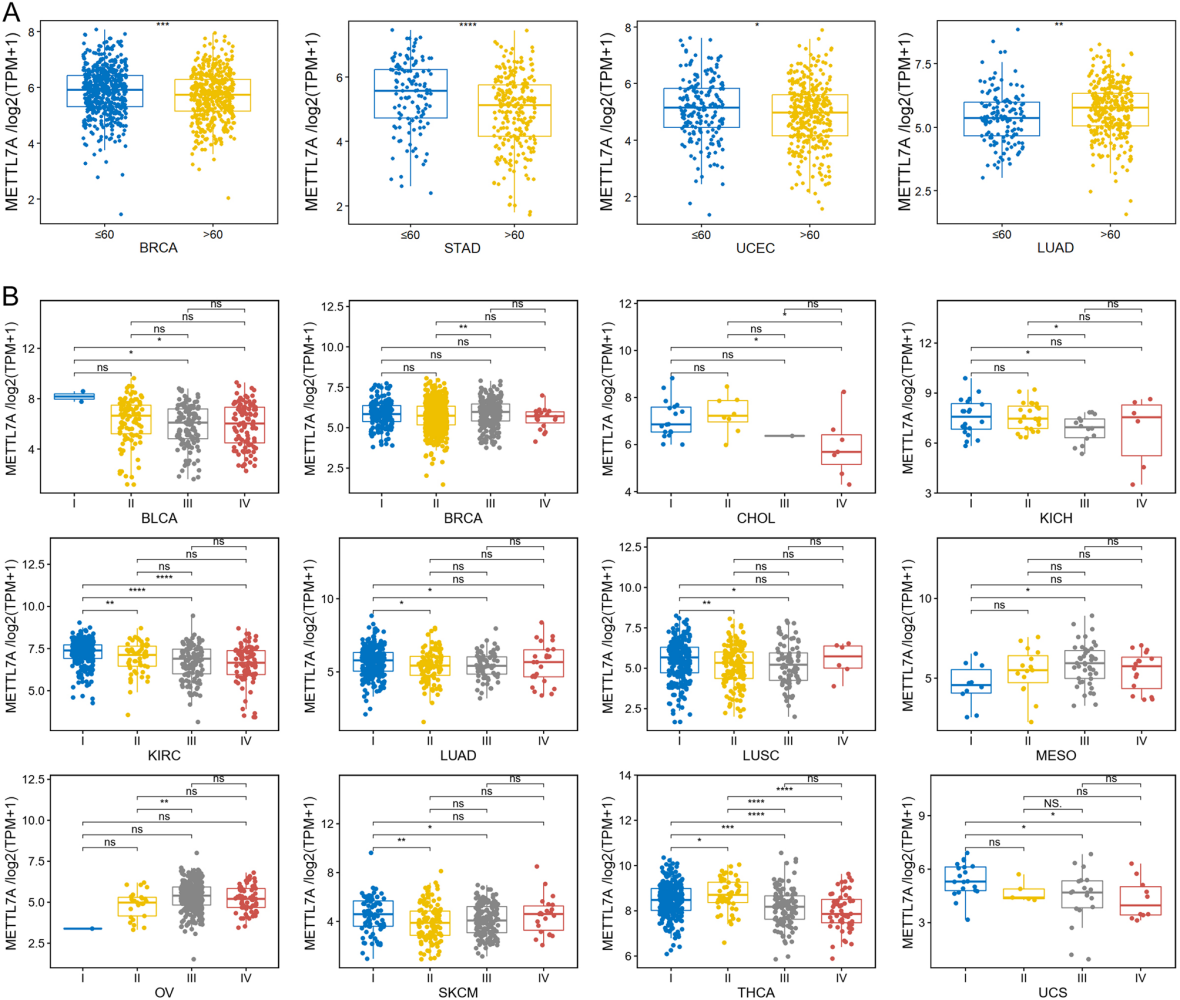
Pan-cancer correlation analysis of METTL7A and clinical phenotypes. (**A**) Patients aged ≤ 60 years showed higher METTL7A expression in BRCA, STAD, and UCEC, while had lower METTL7A expression in LUAD. Wilcoxon test was used. (**B**) METTL7A was significantly correlated with tumor stage in BLCA, BRCA, CHOL, KICH, KIRC, LUAD, LUSC, MESO, OV, SKCM, THCA, and UCS. Data was shown as log_2_(TPM + 1), and Wilcoxon test was used to compare the expression of METTL7A between different groups. *P < 0.05, **P < 0.01, ***P < 0.001, ****P < 0.0001. ns meant no significant difference.

Patients aged ≤ 60 years showed higher METTL7A expression in STAD, BRCA, and UCEC, while had lower METTL7A expression in LUAD. (Fig. 3A). We also assessed the expression difference of METTL7A in different tumor stage. Significant correlation between METTL7A expression and tumor stage was found in BRCA, BLCA, CHOL, KIRC, KICH, LUSC, LUAD, OV, THCA, MESO, SKCM, and UCS (Fig. 3B). These results suggested that METTL7A was significantly correlated with clinical phenotypes of LUAD patients.

### Genetic and epigenetic alterations of METTL7A across cancers

**Figure 4:**
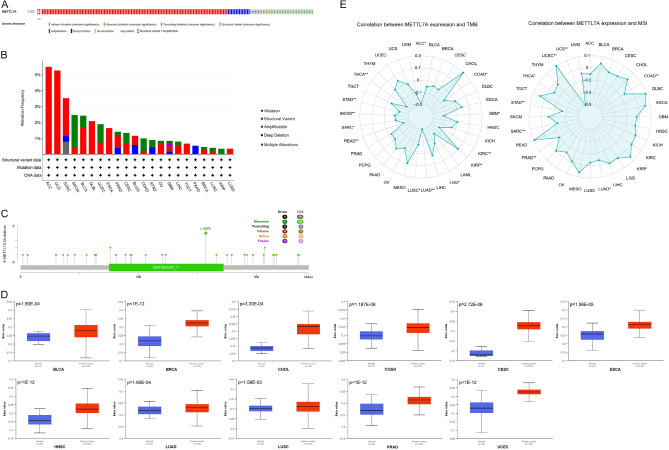
Genetic and epigenetic alterations of METTL7A across cancer types. (**A**) The overall mutation ratio of METTL7A. (**B**) Alterations summary of METTL7A. DLBL, Diffuse Large B-Cell Lymphoma; BLGG, Brain Lower Grade Glioma. (**C**) The mutation sites and number of METTL7A genetic alterations. (**D**) The promoter methylation status of METTL7A was analyzed by UALCAN database. Data was shown as beta value, and Student’s t-test was used to compare the promoter methylation difference between normal and tumor groups. (**E**) Pan-cancer correlation analysis of METTL7A and TMB and MSI. Pearson correlation analysis was used to calculate the correlation between METTL7A expression and TMB or MSI. *P < 0.05, **P < 0.01.

Genetic alteration of METTL7A across cancers was evaluated using the cBioPortal database (https://www.cbioportal.org/). The overall mutation ratio was only 1.3% (Fig. 4A). The highest frequency of METTL7A alteration appeared in ACC, UCS, and SARC (> 3% alteration frequency) (Fig. 4B), and L157F/I was the most frequent missense mutation (Fig. 4C). Amplification, mutation, and deep deletion were the top three types of genetic alteration of METTL7 in cancers. ACC, UCS, and SARC ranked the top 3 types of tumors for METTL7A mutation, however, the highest METTL7A mutation frequency was only 5.49% found in ACC. These results suggest that mutation of METTL7A might not be the main reason of METTL7A expression.

UALCAN (The University of Alabama at Birmingham CANcer data analysis Portal) is a comprehensive online tool for analyzing cancer OMICS data including expression, survival, and methylation^21^. We analyzed the promoter methylation status of METTL7A across cancers using UALCAN (https://ualcan.path.uab.edu/index.html), and found that METTL7A was hypermethylated in 11 type of cancers (Fig. 4D). METTL7A was significantly downregulated in these 11 types of cancers (Fig. 1A). These data suggest that the promoter methylation of METTL7A may result in the down-regulation of METTL7A in several types of tumors including LUAD.

TMB and MSI are promising mutational tumor heterogeneity biomarkers that are predictive for immune checkpoint inhibitors immunotherapy^22^. We assessed the association between METTL7A and TMB and MSI (Fig. 4E). METTL7A showed significant negative correlation with TMB in 12 tumors, including ACC, COAD, GBM, LGG, KIRC, LUSC, LUAD, READ, STAD, SKCM, SARC, and THCA. METTL7A showed significant negative linked with MSI in 7 types of tumors, including COAD, LUAD, PRAD, SARC, STAD, THCA, and UCS. These data indicated that METTL7A significantly correlated with TMB and MSI in LUAD, and METTL7A may affect the antitumor immunity via its association with TMB and MSI in LUAD.

### Pan-cancer correlation with and immune microenvironment

**Figure 5:**
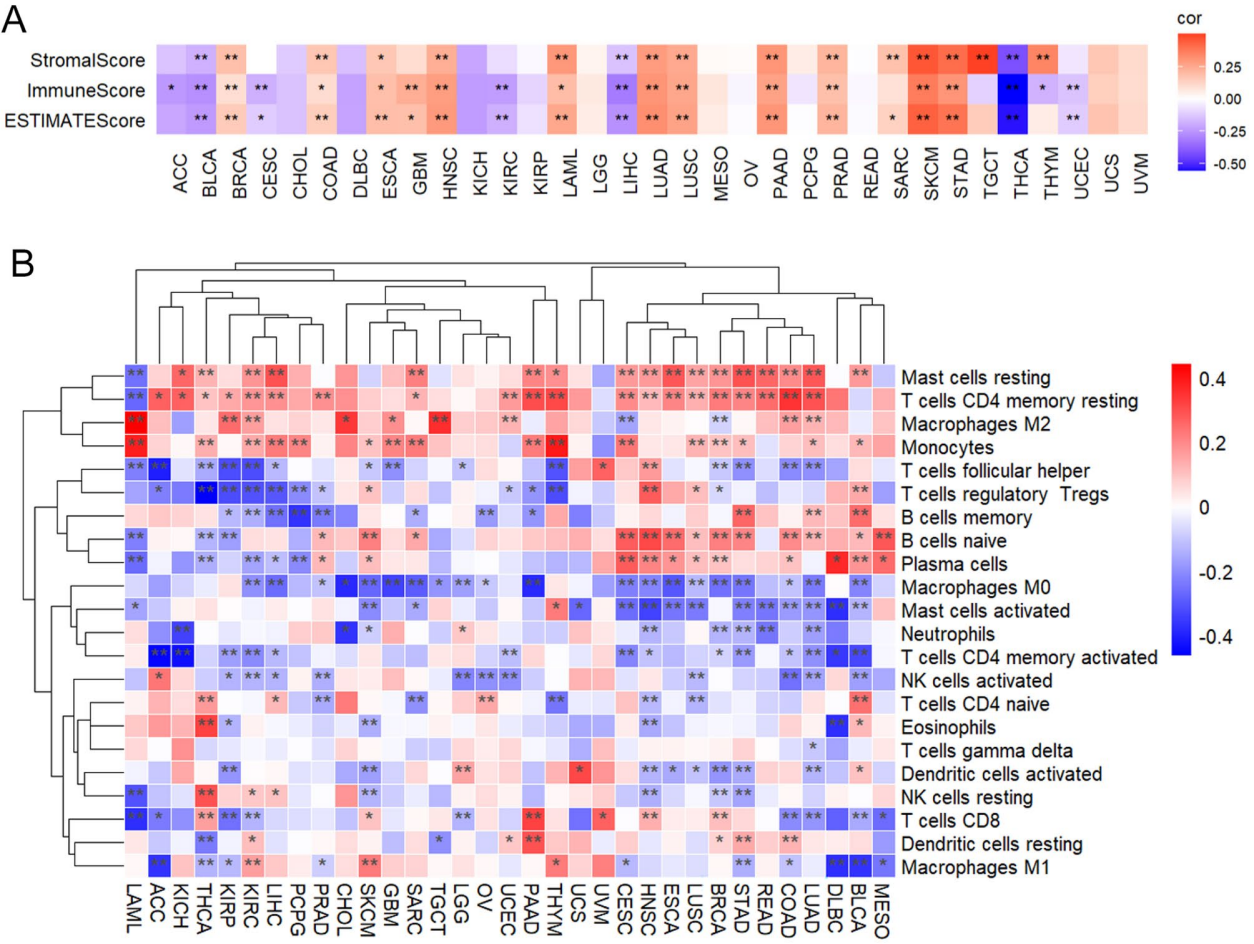
Pan-cancer correlation with and immune microenvironment. (**A**) Heatmap showed the pan-cancer correlation analysis of METTL7A and immune scores. Pearson correlation analysis was used. The heatmap was drawn using our previously published TCGAplot (v4.0.0) R package (https://github.com/tjhwangxiong/TCGAplot). (**B**) The correlation between METTL7A expression and infiltration of 22 types of TIICs were analyzed. Pearson correlation analysis was used to calculate the correlation between METTL7A expression and immune cell ratio. The complete linkage method was used to find similar clusters. *P < 0.05, **P < 0.01. The heatmap was drawn using our previously published TCGAplot (v4.0.0) R package (https://github.com/tjhwangxiong/TCGAplot).

Immune and stromal cells play essential roles in the regulation of development and progression of cancers, accounting for the major components of the tumor microenvironment (TME), and their infiltration levels influence the immunotherapy efficacy. METTL7A was positively linked to immune scores in COAD, BRCA, GBM, ESCA, LAML, HNSC, LUSC, LUAD, PAAD, SKCM, PRAD, and STAD (Fig. 5A). In most tumor samples across the 33 types of tumors, METTL7A was positively correlated with naïve B cells, resting Mast cells, Monocytes, and resting memory CD4 T cells in most tumors, while was negatively associated with Macrophages M0, activated Mast cells, activated memory CD4 T cells, CD8 T cells, and follicular helper T cells (Fig. 5B).

**Figure 6:**
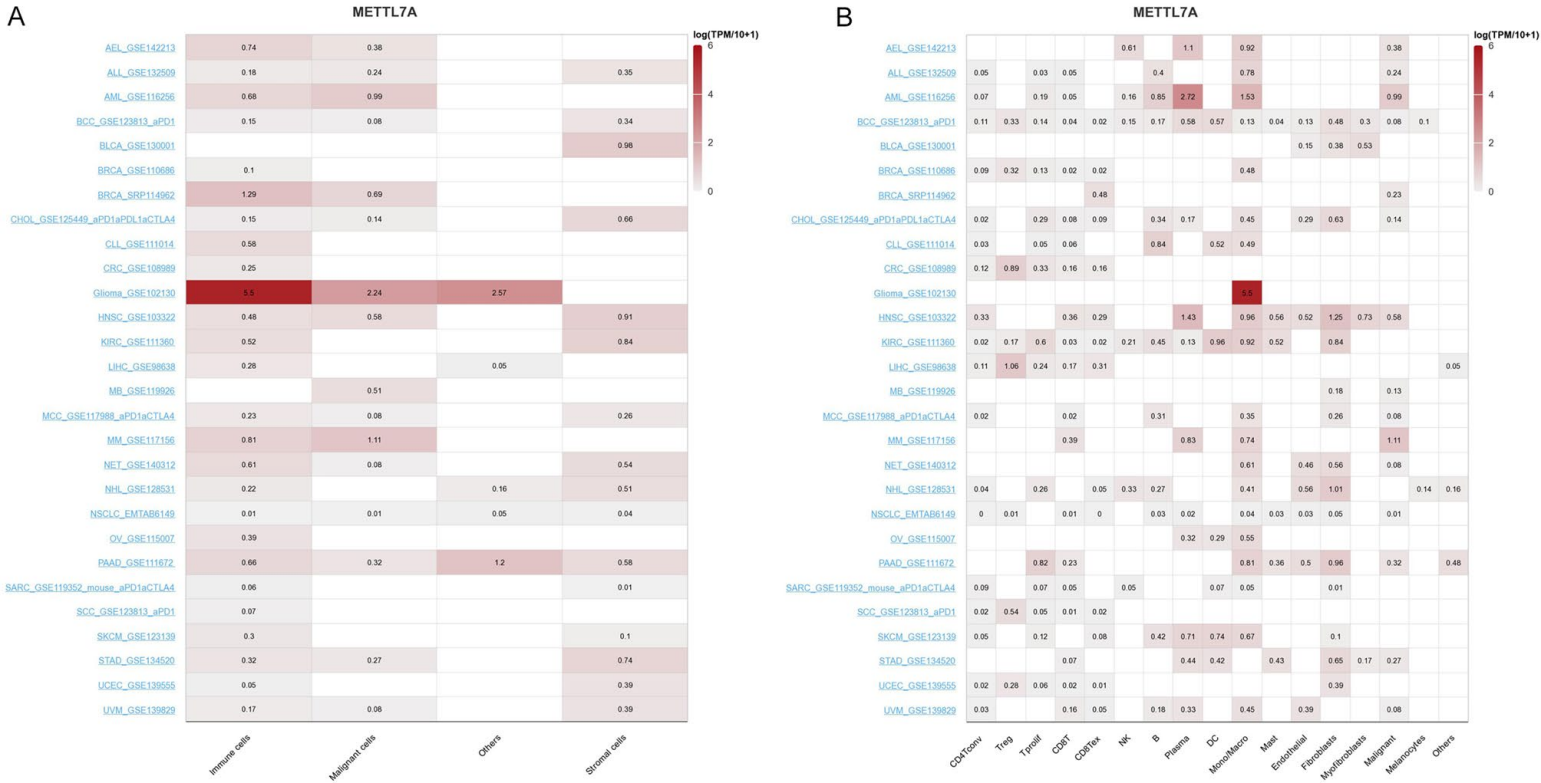
Pan-cancer expression analysis of METTL7A from scRNA sequencing data. (**A**) Pan-cancer expression of METTL7A in immune cells, malignant cells, and stromal cells. (**B**) Pan-cancer expression of METTL7A in subgroups of immune cells.

TISCH2 (http://tisch.comp-genomics.org/home/) is a scRNA sequencing database focusing on TME across different cancer types via providing detailed cell-type annotation and gene expression at the single-cell level. We found that METTL7A was highly expressed in immune cells compared with malignant and stromal cells across several types of tumors (Fig. 6A). In details, METTL7A was highly expressed in Mono/Macro cells and was widely expressed in CD8T cells (Fig. 6B).

**Figure 7:**
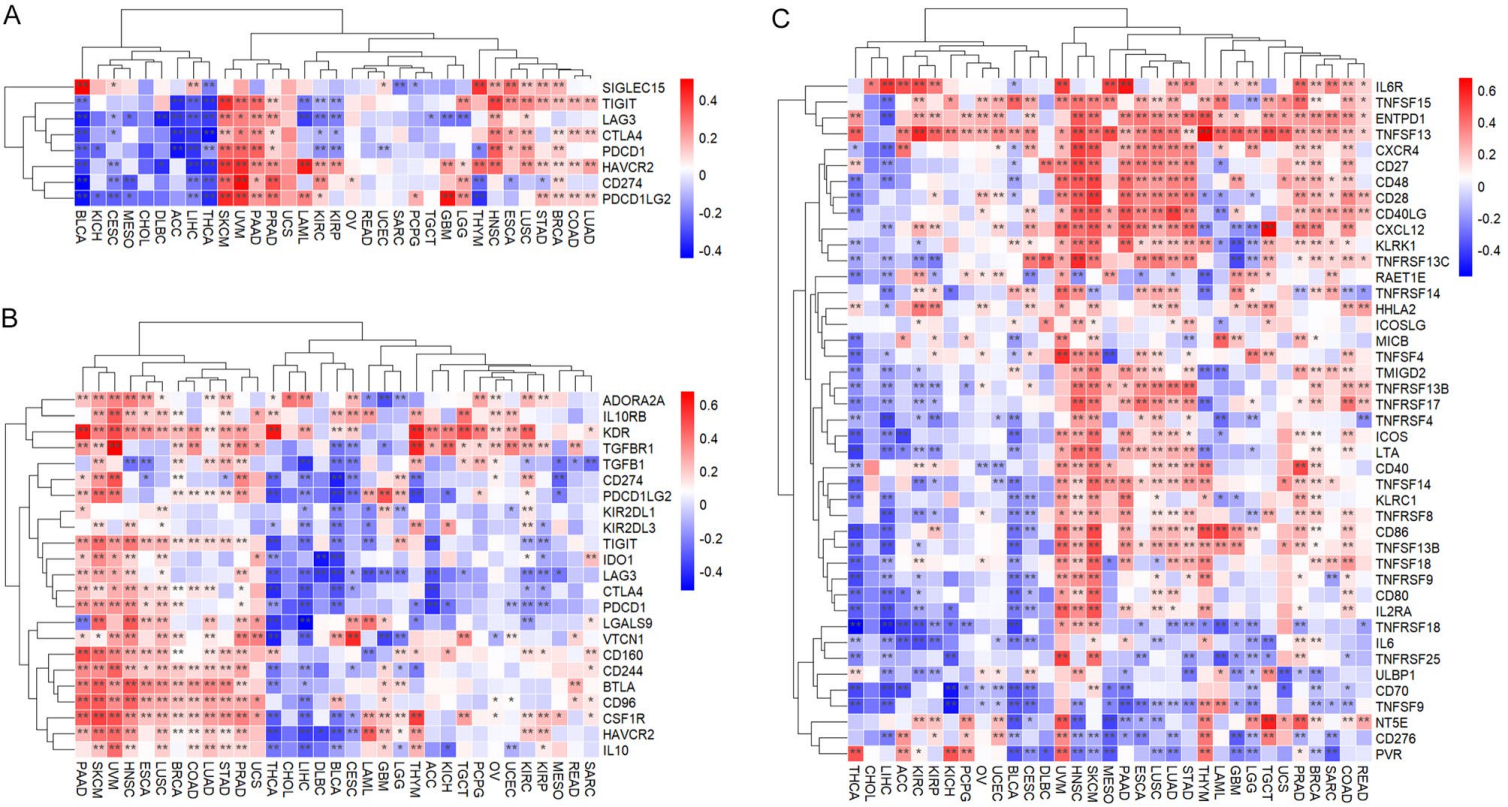
Correlation between the expression of METTL7A with immune related genes. (**A**) Immune checkpoint-associated, (**B**) Immune inhibitory genes, (**C**) Immune stimulating genes. Pearson correlation analysis was used. Pearson correlation analysis was used to calculate the correlation between the expression of METTL7A and immune related genes. The complete linkage method was used to find similar clusters. *P < 0.05, **P < 0.01. The heatmap was drawn using our previously published TCGAplot (v4.0.0) R package (https://github.com/tjhwangxiong/TCGAplot).

METTL7A was positively associated with almost all immune checkpoint-associated genes in COAD, BRCA, HNSC, LUSC, LUAD, PAAD, STAD, PRAD, SKCM, and UVM (Fig. 7A). METTL7A was positively correlated with almost all immune inhibitory or stimulating genes in COAD, BRCA, HNSC, LUSC, LUAD, PAAD, STAD, PRAD, SKCM, and UVM, while was negatively correlated in BLCA, LIHC, and THCA (Fig. 7B,C).

All these data together indicate that METTL7A expression was widely correlated with immunity in cancers and may affect survival through interacting with immune infiltration. This correlation could be negative or positive depending on tumors.

### Pan-cancer GSEA analysis

**Figure 8:**
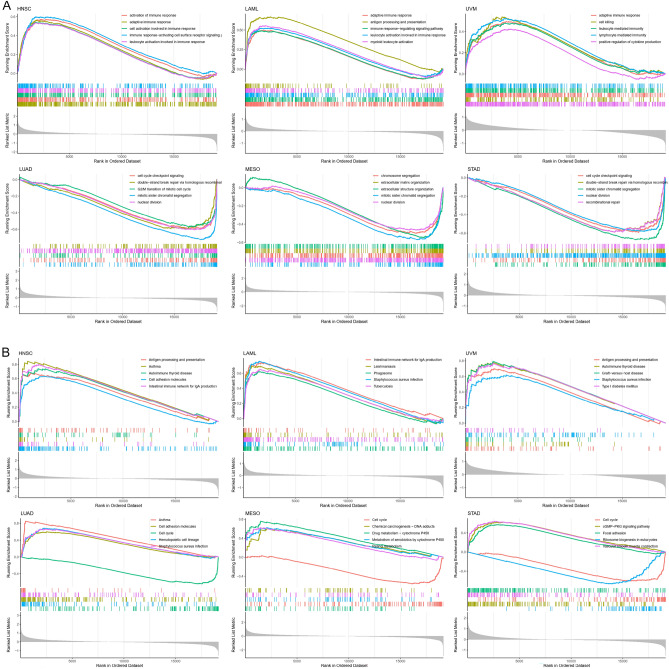
Pan-cancer GSEA analysis. (**A**) GSEA-GO analysis. (**B**) GSEA-KEGG analysis.

We performed GESA-GO and GSEA-KEGG analysis to examine the biological value of METTL7A across tumors. GSEA-GO analysis found that METTL7A was positively correlated with leukocyte activation and adaptive immune response involved in immune response in HNSC, LAML, and UVM, while was negatively correlated with mitotic sister chromatid segregation and cell cycle checkpoint signaling in MESO, LUAD, and STAD (Fig. 8A). GSEA-KEGG analysis revealed that METTL7A was positively associated with antigen processing and presentation in HNSC, LAML, and UVM, while was negatively linked to cell cycle in LUAD, MESO, and STAD (Fig. 8B). These data suggest that METTL7A expression may be involved in the regulation of immune response and antigen processing during tumor development and progression. However, limma, designed for array-based technologies, may not perform as effectively as more specialized packages for bulk RNA-seq analysis, including EdgeR and DEseq2.

### METTL7A expression predicts drug sensitivity

**Figure 9:**
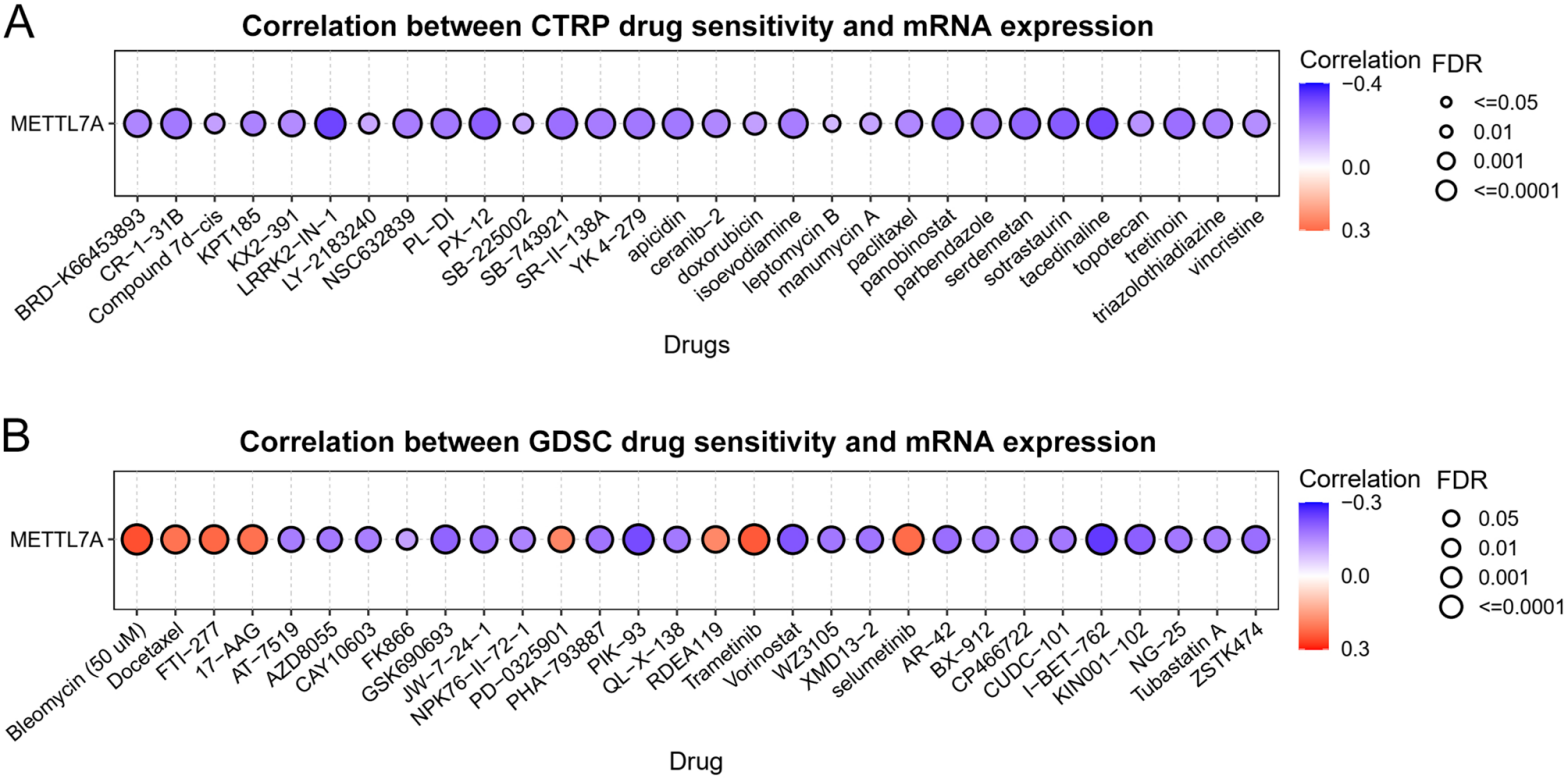
METTL7A expression predicts drug sensitivity. (**A**) The correlation between METTL7A expression and the top 30 CTRP drug sensitivity in pan-cancer was presented. (**B**) The correlation between METTL7A expression and the top 30 GDSC drug sensitivity in pan-cancer was presented.

Correlation between METTL7A and the drug sensitivity in CTRP and GDSC databases was analyzed with Gene Set Cancer Analysis database^23^. Drugs including LRRK2-IN-1 (LRRK2 inhibitor), tacedinaline (inhibitor of the histone deacetylase), GSK690693 (pan-Akt inhibitor), PIK-93 (PI4KIIIβ inhibitor), Vorinostat (inhibitor of the histone deacetylase), and I-BET-762 (BET inhibitor) were negatively correlated with METTL7A (Fig. 9A,B). METTL7A expression may predict the therapeutic effect of these drugs.

### External validation

The above pan-cancer analyses are derived from database. We further performed evaluation with an additional cohort to validate the observed variances of METTL7A across different facets within specific cancer tissues. METTL7A was downregulated in LUAD, and played essential diagnostic and prognostic roles in LUAD. METTL7A acted as a protective role in LUAD (Fig. 1D), highly expressed in LUAD patients aged over 60 years (Fig. 3A), positively correlated with immune scores in LUAD (Fig. 5B). METTL7A showed positive and negative correlation with PDCD1 and CD274 respectively in LUAD (Fig. 7A). BTLA and CD96 ranked the top 2 positively correlated immune inhibitors with METTL7A in LUAD (Fig. 7B). PVR and CD276 ranked the top 2 negatively correlated immune stimulators with METTL7A in LUAD (Fig. 7C). GESA analysis found that nuclear division (Fig. 8A) and cell cycle (Fig. 8B) were enriched in METTL7A low expression LUAD patients.

**Figure 10:**
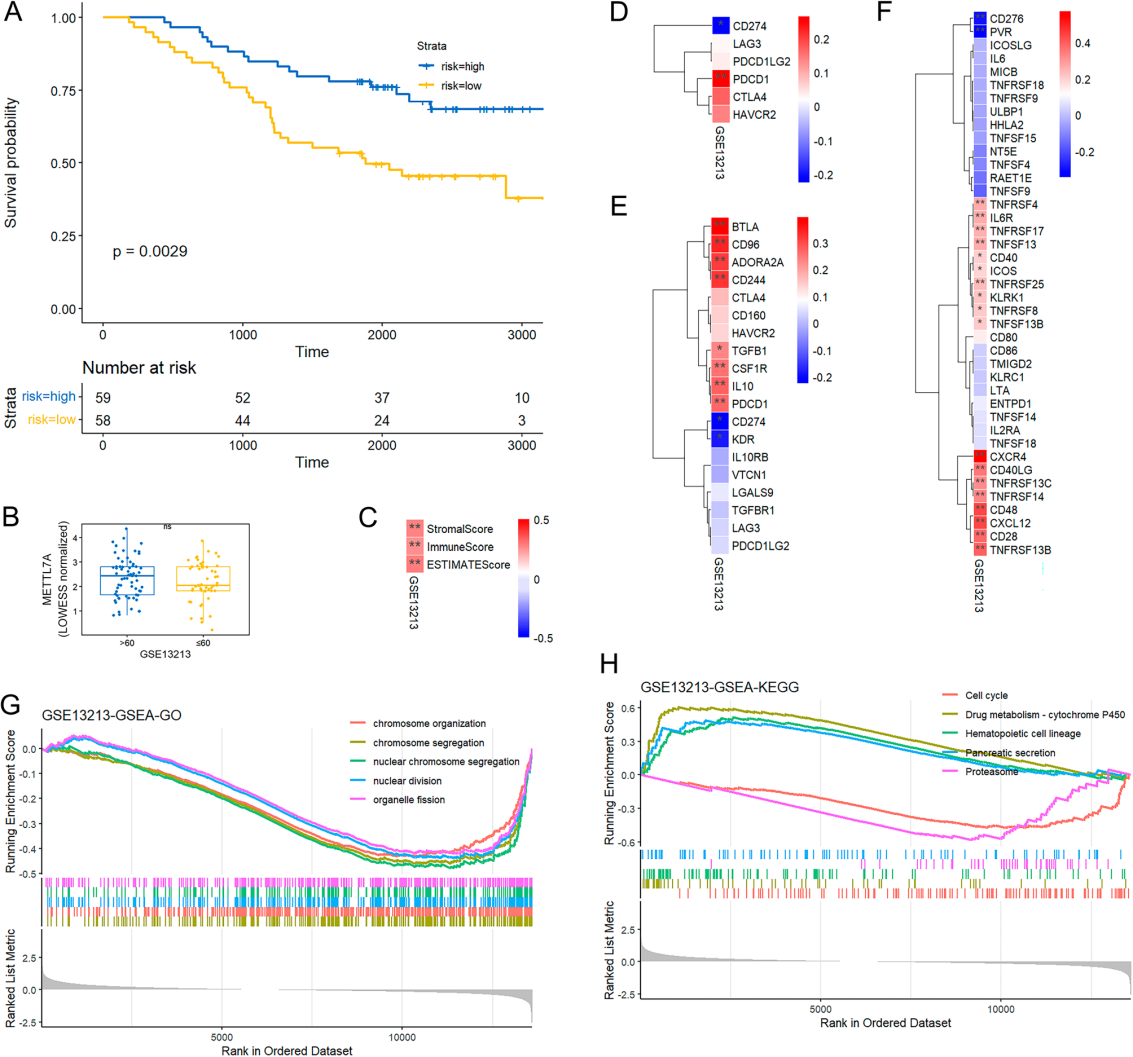
Validation analysis in GSE13213. (**A**) KM analysis investigated the correlation between METTL7A and OS in GSE13213 LUAD patients. (**B**) Expression of METTL7A between patients aged ≤ 60 years and those aged > 60 years. (**C**) Heatmap showed correlation between METTL7A and immune scores in GSE13212 LUAD patients. (**D**) Heatmap showed correlation between METTL7A and immune checkpoint-associated genes in GSE13212 LUAD patients. (**E**) Heatmap showed correlation between METTL7A and immune inhibitor in GSE13212 LUAD patients. (**F**) Heatmap showed correlation between METTL7A and immune stimulators in GSE13212 LUAD patients. (**G**) GSEA-GO analysis. (**H**) GSEA-KEGG analysis. Pearson correlation analysis was used to calculate the correlation between the expression of METTL7A and immune related genes. The complete linkage method was used to find similar clusters. *P < 0.05, **P < 0.01. The heatmap was drawn using our previously published TCGAplot (v4.0.0) R package (https://github.com/tjhwangxiong/TCGAplot).

GSE13213 dataset was downloaded from the Gene Expression Omnibus (GEO) database (https://www.ncbi.nlm.nih.gov/geo/query/acc.cgi?acc=GSE13213), which included 117 LUAD patients (Tables S1, S2). The KM plot showed that higher expression of METTL7A indicated better prognosis (Fig. 10A). Patients aged over 60 years tended to have higher expression of METTL7A than patients aged less than 60 years (Fig. 10B). METTL7A positively correlated with immune scores (Fig. 10C). METTL7A showed positive and negative correlation with PDCD1 and CD274 respectively (Fig. 10D). BTLA and CD96 ranked the top 2 positively correlated immune inhibitors with METTL7A (Fig. 10E). PVR and CD276 ranked the top 2 negatively correlated immune stimulators with METTL7A (Fig. 10F). GESA analysis found that nuclear division (Fig. 10G) and cell cycle (Fig. 10H) were enriched in METTL7A low expression LUAD patients. Results from GSE13213 were consistent with the results from TCGA LUAD cohort considering expression, prognosis, immune scores, and immune-related genes, indicating the robustness of our findings.

## Discussion

In our pan-cancer bioinformatics analysis, we conducted a comprehensive investigation of the multi-faceted features of METTL7A, including its expression, prognosis, genetic mutation, epigenetic alteration, mutational tumor heterogeneity, TME immune cell infiltration, and signaling pathway across human cancers. Our results showed that METTTL7A was significantly decreased in most human cancers, and exhibited good diagnostic performance in 14 types of cancers with an AUC greater than 0.8. Moreover, we found a negative correlation between METTL7A and prognosis, which could be partially explained by the correlation between METTL7A and immune therapy-related genes, immune cell infiltration, TMB, and MSI. To further validate our findings on gene expression in cancers, we performed immunohistochemistry staining and observed that the protein levels of METTL7A were increased in BRCA, UCEC, COAD, PRAD, and KIRC. Overall, our results support the hypothesis that METTL7A plays a crucial role in cancer formation, survival prognosis, and immune therapy across different cancer types.

METTL7A is located on chromosome 12q13.12 and comprises 2 exons. It is believed to be a probable RNA methyltransferase, and its expression could be regulated by the gene body methylation^11^. In this study, we observed increased methylation levels in the promoter region of METTL7A in 11 type of cancers from TCGA, and METTL7A were downregulated in these cancers, suggesting that hypermethylation of METTL7A promoter may contribute to the down-regulation of METTL7A in these tumors. Notably, METTL7A was found to be decreased in THCA, BRCA, and LUAD^10,24^. It was also identified as a potential target for sensitizing choriocarcinoma cells to methotrexate-based chemotherapy^13^.

Genetic alterations were observed in the form of amplification and mutation, with the latter being the highest genetic alteration of METTL7A across human cancers. L157F/I was the most common missense mutation, while the total mutation ratio was only 1.3%. Both genetic and epigenetic alterations could determine the expression level of the target gene. The increased methylation levels and low genetic mutation ratio indicate that the downregulated expression of METTL7A may be primarily caused by epigenetic alteration, suggesting a potential role of METTL7A in the tumorigenesis process.

METTL7A was associated with favorable OS in most cancers, but was identified as a high risk factor in BRCA and LAML through Cox proportional risk model analysis. Furthermore, Patients below the age of 60 exhibited higher expression of METTL7A in STAD, BRCA, and UCEC, whereas those in the same group had lower METTL7A expression in LUAD compared to those aged over 60. Additionally, METTL7A expression tended to be higher in early-stage (I/II) tumors than in advanced-stage (III/IV) tumors. These findings suggest that METTL7A functions as a tumor suppressor and is associated with slower tumor progression, better tumor staging, and may act as a potential early biomarker for both diagnosis and follow-up.

Immunotherapy is gaining much attention in cancer treatment, and both MSI and TMB are essential biomarker of immunotherapy response^25,26^. This study reveals that METTL7A was negatively correlated with TMB and MSI in 13 and 8 types of tumors, respectively, indicating that METTL7A may function to maintain genomic integrity.

Neoplastic tissue acts as a complex organ to build up a microenvironment named TME^27^. TME consists of both stromal and immune cells, which play critical roles in tumor proliferation, invasion, and drug resistance^28^. METTL7A was found to be positively associated with immune scores in 12 types of cancers, while negatively linked to immune scores in BLCA, CESC, LIHC, KIRC, and THCA. Furthermore, METTL7A was increasingly correlated with invasive immune cells in most tumors, including B cells, CD4 T and Mast cells, while negatively correlated with regulatory T (TReg) cells. CD4 T cells enhance the anticancer activity of CD8 T cells and macrophages, and prevent tumor growth^29^. Under normal condition, TReg cells prevent autoimmunity, maintain peripheral tolerance, and limit chronic inflammatory diseases. However, during tumor development and progression, TReg cells suppress sterilizing immunity, inhibit anti-tumor immunity, stimulate tumor growth, promote immune escape, and limit beneficial responses of immunotherapy^30^. These data suggest that the expression of METTL7A was correlated with immune cell infiltration and their immunologic functions in the TME. Moreover, we found that METTL7A was positively associated with immune checkpoint genes in 10 types of cancers, and a similar correlation was found in immune stimulating and inhibitory genes. We also explored the expression of METTL7A at single cell level in scRNA sequencing database TISCH2. We found that METTL7A was highly expressed in immune cells compared with malignant and stromal cells across several types of tumors. In details, METTL7A was highly expressed in Mono/Macro cells and was widely expressed in CD8T cells. These data suggest that METTL7A may control the immune response by affecting the expression of immune-related genes.

We also investigated the biological processes affected by METTL7A. The GSEA-GO analysis indicated that METTL7A may affect tumor pathogenesis via leukocyte activation and adaptive immune response. The GSEA-KEGG analysis indicated the involvement of antigen processing and presentation and cell cycle. The adaptive immune system consists of T lymphocytes, B lymphocytes, and antibodies. Unlike the innate immune system, the adaptive immunity enhances the immune response by the immunological memory initially activated by tumor antigen^31^. The tumor phenotypes may change in response to immune response and evade it, leading to adaptive immune resistance. The immune checkpoint inhibitors therapy such as PD-1 blockade could induce immune response through suppressing adaptive immune resistance^32^. The GO and KEGG analysis showed that the differentially expressed genes between METTL7A high and METTL7A low patients were enriched in antigen processing and presentation, and adaptive immune response, suggesting that METTL7A may play a role in the immune response and immune checkpoint inhibitors therapy.

Drug sensitivity also showed that METTL7A was negatively correlated numerous drugs from CTRP and GDSC databases. These drugs were widely used in clinical practice including inhibitors of LRRK2, histone deacetylase, PI4KIIIβ, and BET. These results suggest that METTL7A may be a good marker for the prediction of the treatment effect of these drugs. However, the underlying mechanisms still require further study.

The pan-cancer analysis of METTL7A also revealed the tumor heterogeneity. For example, COAD and READ, displayed substantial differences in immune scores, despite their anatomical proximity and similar chemotherapy regimens. About 1% of COAD patients harbored METTL7A mutation, and mutation accounted for the majority, while the METTL7A mutation ratio was very low in READ patients. The promoter methylation of METTL7A was significantly higher in COAD patients compared with control subjects, while no significant difference was found in promoter methylation of METTL7A between READ patients and controls. METTL7A negatively correlated with MSI in COAD, while positively correlated with MSI in READ. METTL7A show stronger correlation with immune scores in COAD than READ. These differences may partially contribute to the immune difference between COAD and READ.

METTL7A was downregulated in LUAD, and played essential diagnostic and prognostic roles in LUAD. Moreover, METTL7A was significantly correlated with clinical phenotypes of LUAD patients. High promoter methylation level of METTL7A may result in the down-regulation of METTL7A in LUAD. METTL7A was significantly correlated with TMB, MSI, immune check point genes in LUAD, indicating the antitumor immunity potential of METTL7A in LUAD. Based on these critical findings in LUAD, we further validated our finding in GSE13212 dataset from the GEO database which included 117 LUAD patients. The results from GSE13213 LUAD patients were consistent with those from the TCGA LUAD patients, indicating the robustness of our findings.

In conclusion, we conducted a pan-cancer integrated analysis of METTL7A and found that its expression is significantly decreased in most TCGA cancers. We also observed a correlation between METTL7A expression and clinical prognosis, age, and tumor stage. Our results suggest that METTL7A may act as a favorable prognostic indicator for several types of cancer. Additionally, we found that METTL7A is correlated with TMB, MSI, immune cell infiltration, and immune therapy-related genes in numerous cancers, and its expression varies between different cancer types. Biological validation revealed decreased protein levels of METTL7A in several types of tumors, and overexpression of METTL7A inhibited the invasion of some tumor cell lines. This study has important implications for understanding the function of METTL7A across various cancers and provides a foundation for future accurate individualized immunotherapy. METTL7A may serve as promising diagnostic and prognostic indicator of LUAD.

### Supplementary Information

Supplementary Tables.

## Data Availability

The datasets used during this study can be downloaded from public databases including TCGA and GEO. GSE13213: https://www.ncbi.nlm.nih.gov/geo/query/acc.cgi?acc=GSE13213.

## Abbreviations

m6A: N6-methyladenosine
METTL7A: Methyltransferase-like protein 7A
TCGA: The cancer genome atlas
TMB: Tumor mutational burden
MSI: Microsatellite instability
TPM: Transcripts per kilobase million
ROC: Receiver operating characteristic
AUC: Area under curve
BRCA: Breast invasive carcinoma
UCEC: Uterine corpus endometrial carcinoma
OS: Overall survival
GSEA: Gene set enrichment analysis
GO: Gene ontology
BP: Biological process
KEGG: Kyoto encyclopedia gene and genome
BLCA: Bladder urothelial carcinoma
CESC: Cervical squamous cell carcinoma and endocervical adenocarcinoma
CHOL: Cholangio carcinoma
COAD: Colon adenocarcinoma
ESCA: Esophageal carcinoma
HNSC: Head and neck squamous cell carcinoma
KICH: Kidney chromophobe
KIRC: Kidney renal clear cell carcinoma
KIRP: Kidney renal papillary cell carcinoma
LIHC: Liver hepatocellular carcinoma
LUSC: Lung squamous cell carcinoma
PRAD: Prostate adenocarcinoma
READ: Rectum adenocarcinoma
STAD: Stomach adenocarcinoma
